# Polymyxin B-induced skin hyperpigmentation: a rare case report and literature review

**DOI:** 10.1186/s40360-018-0226-1

**Published:** 2018-07-04

**Authors:** Guanhao Zheng, Li Cao, Zaiqian Che, Enqiang Mao, Erzhen Chen, Juan He

**Affiliations:** 1 Department of Pharmacy, Xiaolan Hospital Affiliated to Southern Medical University, Zhongshan, China; 2 Department of Pharmacy, Zhongshan People’s Hospital, Zhongshan, China; 3 Department of Emergency Intensive Care Unit, Ruijin Hospital Affiliated to Shanghai JiaoTong University School of Medicine, Shanghai, China; 4 Department of Emergency Intensive Care Unit, Ruijin Hospital Affiliated to Shanghai JiaoTong University School of Medicine, EICU 3 Floor, 5 Building, 197 Ruijin No. 2 Road, Huangpu District, Shanghai, China; 5 Department of Pharmacy, Ruijin Hospital Affiliated to Shanghai JiaoTong University School of Medicine, 202 Room, 12 Building, 197 Ruijin No. 2 Road, Huangpu District, Shanghai, China

**Keywords:** Polymyxin B, Skin hyperpigmentation, Sepsis, Case report

## Abstract

**Background:**

Polymyxin B (PMB), which is regarded as the ultimate antibacterial treatment against some intractable gram-negative bacteria with its outstanding anti-bacterial activities, inflicts several adverse effects on patients. However, skin hyperpigmentaion (SH) induced by PMB is very rare. Here, we report a case of polymyxin B-induced skin hyperpigmentation (PMB-iSH) in a 21-year-old female. To the best of our knowledge, this is the first case of PMB-iSH in China.

**Case presentation:**

A 21-year-old female patient with sepsis received the administration of PMB by intravenous injection for the treatment of multi-drug resistant *Klebsiella pneumoniae* (MDR-KP) infection. She later suffered from a rare adverse drug reaction (ADR), namely PMB-iSH, after 5-day PMB administration during her treatment. There were multiple red rashes spread on the whole body skin at first. With the rashes fading away, SH with dark round spots appeared, associated with no pain or pruritus. The skin of the head and neck was darkened evidently, and dark brown spots were spread on the skin of trunk and limbs. About a month after her admission, urged by the relatives, the patient was transferred back to the local hospital for further treatment in the end, and her skin color didn’t recover to the previous state at that time.

**Conclusion:**

Both our case and the literature review highlight that PMB can give rise to SH indeed. Clinicians and pharmacists should attach great importance to this rare pigmentary disorder and further investigation is warranted.

## Background

Skin hyperpigmentation (SH) is a common type of skin disease with various inducing factors. Drug-induced skin hyperpigmentation (DiSH) is an important part of SH, accounting for 10 to 20% of all acquired SH cases [1]. A variety of drugs can trigger DiSH, such as tetracycline, NSAIDs, anti-malarial agents, anti-psychotic agents, cytotoxic agents, amiodarone, etc. [1]. Minocycline is the most common trigger among the aforementioned drugs [2]. Recently, SH has been observed in several patients who received the treatment of intravenous polymyxin B (PMB). Here we discuss a patient with sepsis who was treated by intravenous PMB and suffered from a rare adverse drug reaction (ADR) afterward, namely polymyxin B-induced skin hyperpigmentation (PMB-iSH), on the basis of a literature review of its epidemiology, pathological mechanism, treatment options and prognosis.

## Case presentation

### History and previous admissions

This was a rare case of PMB-iSH in a 21-year-old female in China. In her postpartum period, the patient suffered from chest pain, fever and even coma for a fortnight. She was sent to the local hospital due to cardiac arrest by 4 times on 27th January 2017. After CPR, she regained consciousness gradually but still was in a continuous febrile state. *Klebsiella pneumoniae* was isolated from the samples of blood and sputum cultures. Besides, anti-microbial therapy hadn’t worked effectively since she was treated with cefepime, imipenem and tigecycline.

### EICU admission

The patient was soon admitted to the emergency intensive care unit (EICU) of Ruijin Hospital affiliated to Shanghai Jiao Tong University on 26th April. Upon admissionto our hospital, she was still in fever, unconscious in a dyspneic state, and mechanical ventilation was initiated after tracheotomy with metal tracheal tube.

### Examination in EICU & adjustment for anti-infectious therapy

A full-body computed tomography (CT) scan identified thickened pericardium, bilateral pleural effusion with multiple exudative focuses, hepatosplenomegaly and pelvic effusion in this patient. Empiric antibiotic treatment was started for *Klebsiella pneumoniae* infection with piperacillin-tazobactam (4.5 g, intravenously, q.8 h). The sample of microbial sputum culture on 29th April revealed that a large amount of multi-drug resistant *Klebsiella pneumoniae* (MDR-KP) grew, which was merely susceptible to tigecycline, sulfamethoxazole (SMZ) and PMB. The infection parameters from laboratory examination increased remarkably: hypersensitive C-reactive protein 37.0 mg/L [0~ 3 mg/L] and procalcitonin 3.37 ng/mL [< 0.50 ng/mL]. In light of the above-mentioned examination results, we replaced piperacillin-tazobactam with meropenem (2 g, intravenously, q.8 h) and tigecycline (100 mg intravenously q.12 h) with the addition of colistin (1-million unit by aerosol inhalation q.8 h) on 4th May.

### Occurrence of PMB-iSH

**Fig. 1:**
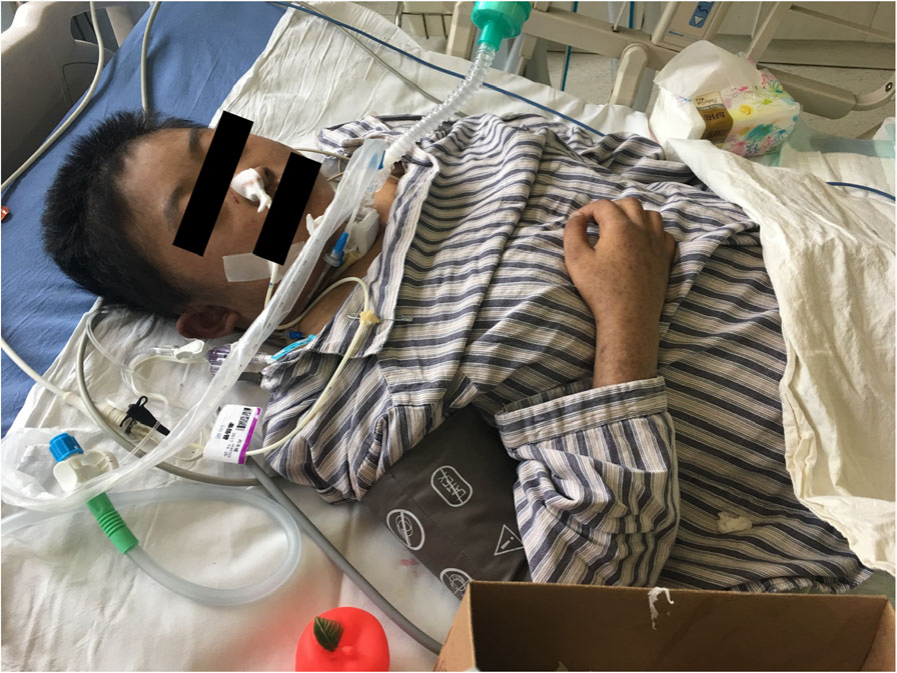
Hyperpigmentation in the head and face

**Fig. 2:**
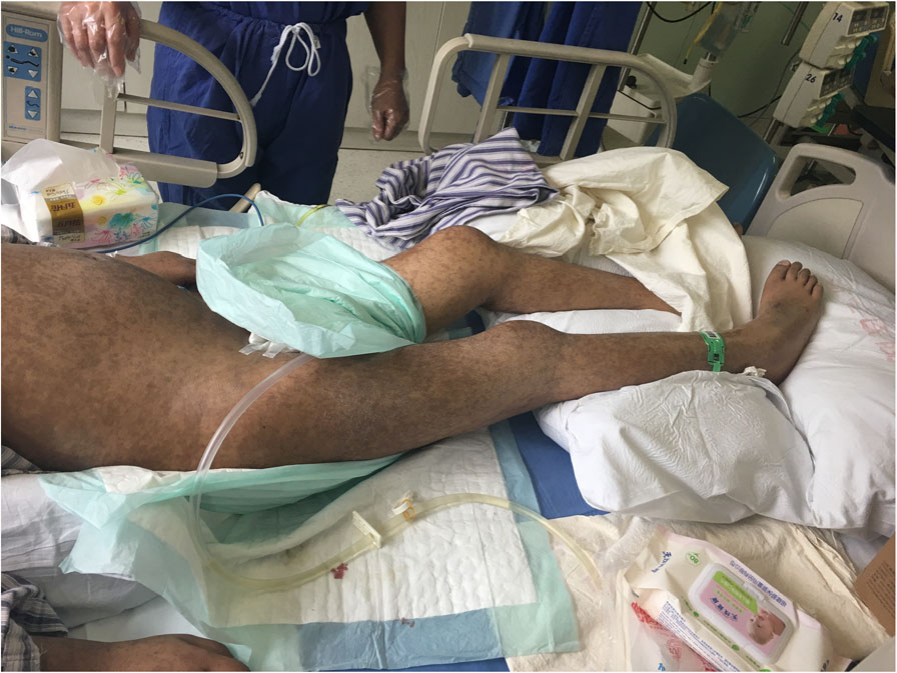
Hyperpigmentation in the lower abdomen

**Fig. 3:**
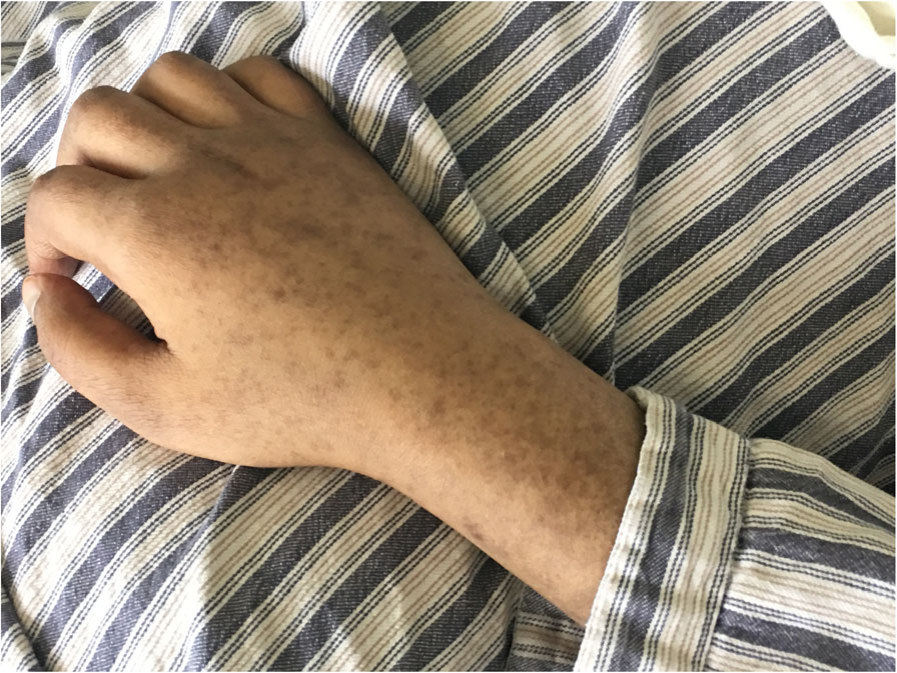
Hyperpigmentation in the right hand

**Fig. 4:**
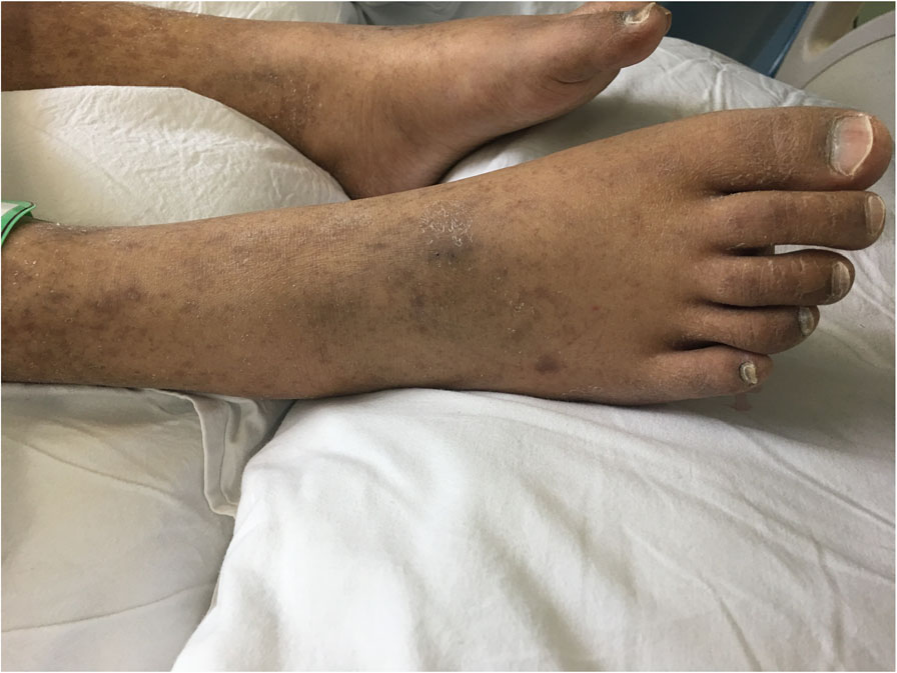
Hyperpigmentation in the right foot

After the adjustment for the treatment, her body temperature dropped to normal and remained stable. During this period, repeated cultures of blood, sputum, vaginal secretion and fluid drained from the hip joint were carried out. MDR*-*KP, susceptible to tigecycline, SMZ and PMB was detected in all the samples. On 18th May, multiple exudations were aggravated in bilateral lungs compared to that upon admission, according to a chest CT scan. As a result, PMB (500,000 units, intravenously, q.12 h) was administered at once. Four days later, there were multiple red rashes spread on the whole body skin. With the rashes fading away, SH with dark round spots was seen on 23rd May (5 days after intravenous PMB) without pain or pruritus. More dark brown spots were spread on the skin of trunk and limbs, especially on that of lower abdomen, right hand and right foot as depicted in Figs. 1, 2, 3 and 4, although the skin of the head and neck was also darkened evidently. Despite this adverse event, the therapeutic regimen was still applied in consideration of MDR*-*KP infection of multiple organs. PMB was utilized in the same dosage until the patient was transferred back to the local hospital, and skin biopsy wasn’t performed.

### Post-treatment course

**Fig. 5:**
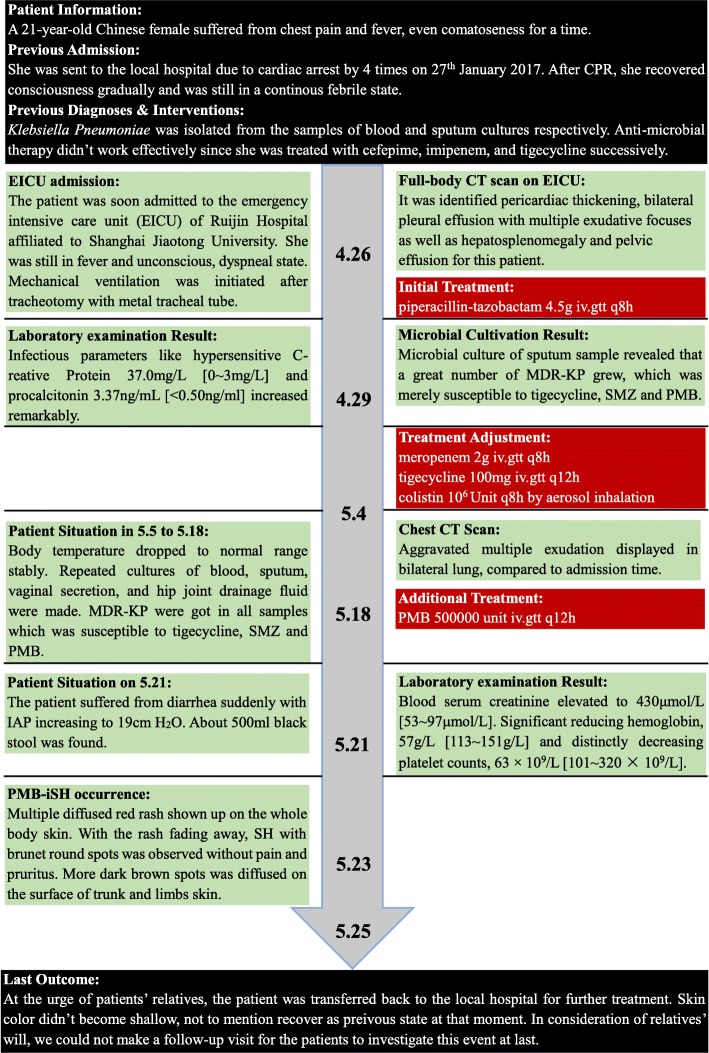
Treatment timeline of this patient

On 21st May, she suffered from diarrhea suddenly with intra-abdominal pressure (IAP) increasing to 19 cm H_2_O. About 500 ml black stool was found in the patient’ s excrement. Her serum creatinine was elevated dramatically to 430 μmol/L [53~ 97 μmol/L], and anuria occurred. The laboratory examinations revealed the following results: the level of hemoglobin was significantly reduced to 57 g/L [113~ 151 g/L] and platelet counts 63 × 10^9^/L [101~ 320 × 10^9^/L]. Her liver function was impaired moderately: total bilirubin was 43.6 μmol/L [4.7~ 24 μmol/L], direct bilirubin was 26.0 μmol/L [0~ 6.8 μmol/L], and γ-GT went up to 103 IU/L [7~ 64 IU/L]. The results of coagulation test were as follows: APTT 50.1 s [22.3~ 38.7 s], PT 12.9 s [10.0~ 16.0 s], TT 24.80s [14.00~ 21.00s], Fg 1.5 g/L [1.8~ 3.5 g/L], FDP 12.5 mg/L [0~ 5 mg/L] and D-Dimer 2.66 mg/L [< 0.55 mg/L]. They indicated that the patient suffered from coagulation abnormality and hyperfibrinolysis, hence disseminated intravascular coagulation (DIC). After phlebotomy, the transfusion of cryoprecipitate prepared from plasma frozen within 24 Hours (PF24) started immediately. Meanwhile, low-dose heparin (3 U/kg/h) was administered prudently for anticoagulation therapy. 2 days later, bleeding disappeared. On 25th May, urged by the relatives, the patient was transferred back to the local hospital for further treatment. At that time, her skin color didn’t recover to the previous state. Owing to her relatives’ will, we failed to make a follow-up visit. The treatment timeline is shown in Fig. 5.

## Discussion & conclusion

Of polymyxins and lipopeptide antibiotics, colistin and PMB were isolated from Paenibacillus ploymyxa and has been available in clinical use since 1959. They have excellent anti-microbial activity against a variety of gram-negative bacteria. Unfortunately, the polymyxins were once replaced by those more effective and safer antibiotics like aminoglycosides on account of their severe nephrotoxicity and neurotoxicity. But in the last few decades, great importance was attached to the polymyxins again by clinicians due to the inevitable appearance of multi-drug resistant (MDR) bacteria. They were even regarded as the ultimate antibacterial treatment against some intractable gram-negative bacteria with their outstanding anti-bacterial activity as well, such as *Escherichia coli*, Pseudomonas aeruginosa, Acinetobacter baumannii, carbapenem-resistant Enterobacteriaceae, etc. [3–5].

When it comes to the polymyxins, their chemical structure, antimicrobial spectrum and mechanism are extremely similar, although colisitin is a pro-drug for clinical use [4]. The most common ADR of the polymyins is nephrotoxicity, while neurotoxicity and hypersensitivity are also other main ADRs [6]. It is a nonnegligible fact that SH is proved as a significant side effect induced by PMB, and no SH cases induced by colistin have been reported.

Only a few case reports on PMB-iSH have been found so far. Knueppel R.C. et al. reported two suspected cases in 2007 for the first time [7]. A patient who suffered from MDR-KP infection was found with SH after treated by intravenous PMB for 4 days, according to the description by Lahiry S. et al. [8]. Besides, there were several cases in neonates and infants as well. Gothwal S. et al. reported 3 babies who had progressive SH after intravenous PMB. They maintained that the cumulative effect of PMB might be a possible reason for darkened skin, which was likely to cause the immaturity of kidney functions of neonates, since PMB was excreted through the kidney mainly [9]. Interestingly, Shih L K et al. used Felix von Luschan Skin Color Chart to classify the color of skin tone into 36 different scales. 16 infants were recruited in this study, treated by 15,000 units/kg intravenous PMB every 12 h for 14 days. Based on the average change of skin tone, the skin tone of all the subjects were progressively darkened during the treatment period and reached the peak at the end of the treatment of intravenous PMB (on the 14th day) [10].

In 2016, Mattos K.P. et al. carried out a cohort study on PMB-iSH, which included 60 patients with gram-negative infection who received a 14-day PMB treatment. PMB-iSH was found in 15% of the subjects usually on the 3rd day of the treatment period. Their skin color were darkened to various shades of brown, particularly in the neck and face [11]. It was reported in another study that this pigmentary disorder was found in 8% of 249 patients who used PMB [12].

To the best of our knowledge, the case previously presented is the first case of PMB-iSH in China. On the basis of Naranjo Adverse Drug Reaction Probability Scale, PMB was probably associated with SH (+ 1 for conclusive reports on this reaction, + 2 for adverse events occurring after PMB administration, + 1 for confirmed by objective evidences, + 2 for the absence of possible alternative causes = 6 points) [13, 14].

Besides PMB, the patient received omeprazole, meropenem, SMZ, colistin, tigecycline and heparin during her treatment period. Among these drugs, tigecycline and omeprazole could cause DiSH. It was reported that tigecycline could induce SH, similar to minocycline [15]. However, SH occurred after the use of PMB for a few days, and their temporal correlation was found in this case. Hence, we considered PMB was the trigger of SH rather than tigecycline and omeprazole. In addition, drug-drug interaction should not be ignored. The combination of colistin and PMB may increase the risk of nephrotoxicity, which may cause the cumulation of PMB in vivo. Due to the limited medical resources and the critical condition of this patient, no further treatment was carried out for this issue.

However, it must be pointed out that acute renal insufficiency may be an important factor for PMB-iSH. Since up to 60% of PMB is excreted unchanged with urine and strongly associated with nephrotoxicity, its elimination half-life increases with reduced renal function. For individuals with renal impairment, reduced dose of PMB is required because of lower clearance rate. Back to this patient, her glomerular filtration rate (GFR) was almost 14.44 mL/min, indicating she suffered from severe acute kidney injury (AKI) during her treatment period. This could give rise to PMB accumulation so that the blood concentration would significantly increase. Although there is no direct evidence to prove that PMB-iSH is a dose-dependent ADR, higher drug level in the body can be a related factor for skin darkening in some studies [9, 10].

The pathological mechanism of PMBiSH is still unclear. Overall, there are 4 possible basic pathological mechanisms of DiSH: (1) the most common drug-induced mechanism is the production and accumulation of melanin within cutaneous cells,, especially in dermal macrophages; (2) the druge accumulation, associated with no melanin, usually exists within extracellular matrix or within dermal macrophages; (3) a specific drug plays a key role in the synthesis of other special pigments like lipofuscin; (4) drug-associated injury to dermal vessels usually results in the leakage of many red blood cells and the deposition of iron [1]. Most of relevant researches maintain that DiSH is attributed to an extensive inflammation, induced by inflammatory factors and producing allergic or toxic effects.

One probable mechanism of PMB-iSH is that PMB induces the release of histamine and the synthesis of melanin [11, 16, 17]. Histamine is released by basophils, mast cells and neurons in skin tissues, which can activate the inflammatory reaction by acting on 4 receptors (H_1_ to H_4_) [18]. Yoshida M. et al. described the melanogenic effect of histamine: it could activate the H_2_ receptor of melanocytes, then up-regulate the activities of both tyrosinase and protein kinase A with the latter acting as a key role in melanogenesis [19]. Furthermore, it is proved that pigmented skin is more common in the head and neck since there are more melanocytes distributed in them [12].

Another presumed mechanism is related to epidermal langerhans cells. The research by Miori L. et al. revealed that the occurrence of SH meant the patient was at the stage of the post-inflammatory pathological progress, and the langerhans cells played a crucial role in chronic inflammatory skin diseases [20]. According to the histopathological results of a PMB-iSH patient’s skin biopsy, Mattos K. P. H. et al. found that the greater proliferation of epidermal langerhans cells and dermal dendritic cells occurred indeed [12]. Based on the information above, it is presumed that PMB is an initiating factor of the pathological progress.

Last but not least, it was described by Choi H. et al. that IL-6 could inhibit the proliferation of melanocytes and the generation of melanin [21]. Mattos K. P. H et al. found the expression of IL-6 was lower in several patients who suffered from the pigmented skin with PMB [12]. These research results may link the level of IL-6 to PMB-iSH. Further investigations should be carried out for verification.

The treatment of DiSH has already been challenging, let alone that of PMB-iSH. No specific treatment has been reported in literature up to now. This may be attributed to the fact that the illness is chronic and continuous progression that can easily relapse. For the patients whose symptoms can be relieved by lowering dosage, it is of great importance to find the balance between DiSH and therapeutic effects. What’s more, it is necessary to find suitable alternative drugs and adjust the dosage in accordance with the patient’ s physiological conditions and other clinical parameters. Another intervention is to avoid the exposure to sunlight, particularly ultraviolet ray, as much as possible so as to reduce the synthesis and accumulation of melanin. Topical or laser therapy through skin whitening agent may also be alternative ways to help the patients whose melanin concentrate within epidermal cells get rid of DiSH [1, 2].

Nowadays, it is still controversial on whether ceasing PMB can reverse the pathological change to pigmented skin and restore the original color. Zavascki A. P. et al. treated a 55-year-old male patient with hospital acquired pneumonia (HAP) by 14-day intravenous PMB treatment. His skin of the head and neck was evidently darkened during the treatment period. His skin tone was not restored to normal 3 months after PMB was withdrawn. On the basis of the Naranjo Adverse Drug Reaction probability scale, PMB was probably associated with hyperpigmentation (+ 2 for adverse events occurring after drug administration, + 1 for confirmed by objective evidences, + 2 for the absence of possible alternative cause = 5 points) [22]. A 14-year-old female patient with aplastic anaemia who experienced an allogenic haematopoietic stem cell transplantation also suffered from PMB-iSH. On the 5th day of PMB treatment to treat postoperative infection, there was diffused SH and dark round spots on her upper body. Skin biopsy displayed that the patient underwent interface dermatitis with vacuolar damage. There were lots of melanophages and melanin pariticles within epidermal and dermal cells. Unlike the former patient, this patient was found with gradual recovery at the 3-month follow-up visit. Her skin color was restored to normal and the dark spots got shallow. And the spots almost disappeared at the 6-month follow-up visit [23].

There are some novel findings from our case. First of all, for the first time, it is observed that the appearance of red rashes is prior to the occurrence of PMB-iSH. We may infer that PMB-iSH is possibly related to drug-induced hypersensitivity reaction, which is caused by the release of histamine and characterized as a symptom of rash and erythra. Secondly, based on our literature review, PMB-iSH mostly occurs on the skin of the head, neck and upper body. But in our case, not only did SH occur in the aforesaid locations, but also more serious symptoms were seen on the skin of the lower body and limbs.

In conclusion, PMB can give rise to SH indeed. Both clinicians and pharmacists should attach great importance to it. With the pathological mechanism still unclear, one of its probable reasons is associated with the release of histamine. Once PMB-iSH was observed, we should weigh the advantages and disadvantages of using PMB, and keep balance between curative effects and adverse event by such measures as dosage adjustment, cessation of drug administration and change to alternative antibiotics. Additionally, topical therapy and laser therapy can be treatments for PMB-iSH. Overall, further investigation is warranted.

## Abbreviations

ADR: Adverse drug reaction
AKI: Acute kidney failure
CPR: Cardiopulmonary resuscitation
CT: Computed tomography
DiSH: Drug-induced skin hyperpigmentation
EICU: Emergency intensive care unit
GIB: Gastrointestinal bleeding
HAP: Hospital acquired pneumonia
IAP: Intra-abdominal pressure
IL-6: Interleukin-6
MDR: Multi-drug resistance
MDR-KP: Multi-drug resistant *Klebsiella pneumoniae*
PMB: Polymyxin B
PMB-iSH: Polymyxin B-induced skin hyperpigmentation
SH: Skin hyperpigmentaion
SMZ: Sulfamethoxazole
